# The effect of short foot exercise using visual feedback on the balance and accuracy of knee joint movement in subjects with flexible flatfoot

**DOI:** 10.1097/MD.0000000000019260

**Published:** 2020-03-27

**Authors:** Ju Sang Kim, Mi Young Lee

**Affiliations:** a Department of Physical Therapy and Rehabilitation, Yeungnam University Hospital, Daegu; b Department of Physical Therapy, College of Biomedical Science, Daegu Haany University, Gyeongsansi, Republic of Korea.

**Keywords:** flexible flatfoot, short foot exercise, visual feedback

## Abstract

**Background:**

Flexible flatfoot is a condition characterized by the deformations of the foot where the calcaneus is pronated by weight support. Flat feet can affect balance and the entire chain of motion, causing indirect problems in adjacent joints. We investigated the effects of short foot exercise (SFE) using visual feedback on the static balance and function of proximal joints in subjects with flexible flat feet.

**Method and analysis:**

This study involved 30 participants who were assigned to either of the 2 groups: the flexible flatfoot group (n = 15, 8 men and 7 women, aged 22.00 ± 2.07 years) and normal foot group (n = 15, 7 men and 8 women, aged 22.13 ± 1.55 years). All subjects performed the SFE with visual feedback. SFE programs were performed 20 minutes a day, 5 times a week, for a total of 5 weeks. The static balance and accuracy of knee joint motions were compared before and after training.

**Results:**

There was a significant difference in static balance pre- and post-exercise in the flatfoot group but not in the normal foot group. Moreover, in the flatfoot group, the accuracy of knee joint motions was significantly different between pre- and post-exercise in the closed chain but not in the open chain.

**Conclusion:**

This study examined the influence of SFE using visual feedback on the balance and accuracy of knee joint movements in subjects with flatfoot and demonstrated that this exercise, using visual feedback, improved the balance and accuracy of knee movement.

## 1 Introduction

Flexible flatfoot is a condition characterized by the deformations of the foot where the calcaneus is pronated by weight support. The weight is also distributed to the inside of the foot, causing the medial longitudinal arch (MLA) to go downward or even disappear. Additionally, excessive mobility of the middle part of the foot requires more effort to maintain posture control and stability of the foot.^[1]^ The weakening of the soles of the plantar fascia reduces the ability to disperse impact shock, and excessive compensation of external muscles causes fatigue that can lead to overuse syndrome.^[2]^ Flat feet may cause functional instability of the foot, affecting the entire kinetic chain, as well as balance and proprioception. This can indirectly lead to various problems in the proximal body part such as knee joint, hip joint, and spine.^[3,4]^

A variety of clinical interventions are available for the treatment of flat feet, such as orthopedic surgery, orthotics, and taping. In particular, short foot exercise (SFE) is commonly used as a therapeutic exercise to strengthen intrinsic foot muscles. SFE is a training used to create an MLA by pulling the first metatarsal bone head to the heel without bending or excessively extending the toes. Mulligan and Cook suggested that SFE may be helpful in improving the balance in functional movement of both, normal and flat feet subjects, preventing navicular drop (ND) through intrinsic muscle activation.^[5]^ Also, SFE can be used to further activate abductor hallucis (AbdH) supporting navicular stability, increasing the stability of foot.^[6]^ It is difficult to perform the exact SFE without the compensatory movements of the extrinsic muscles, such as the tibialis anterior, strengthening the intrinsic muscle selectively. Therefore, we suggest that SFE may require training with feedback to prevent compensatory movements and achieve correct posture for flexible flat feet.

Feedback provides correct posture, motivation, and facilitates communication of the central nervous system,^[7]^ including verbal, visual, auditory, and ultrasound image feedbacks.^[8–12]^ Visual feedback is commonly used for balance training in stroke patients and to determine the level of central nervous system function with motion accuracy.^[13,14]^ Additionally, visual feedback is used to stimulate spinal stabilization muscles, such as pelvic movements, core muscle movements, and motor control training by recognizing the degree of feedback with the instrument panel through pressure of the air cushion to the subjects with pain in the back and neck.^[15,16]^

There have been many studies on the methods of interventions, including insole, taping, and SFE for flat feet subjects. However, only few studies have been conducted to identify the effects of SFE with visual feedback. In the current study, we investigated the effects of SFE using visual feedback on the static balance and function of proximal joint in subjects with flexible flat feet.

## 2 Method

### 2.1 Study design

This study was a prospective non-randomized controlled trial consisting of 2 groups: The flexible flatfoot group and normal foot group. The study protocol was conducted in accordance with the principles of the Declaration of Helsinki and approved by the Institutional Review Board of Daegu Oriental Hospital of Daegu Haany University (DHUMC-D-17017-PRO-02).

### 2.2 Participants

A total of 48 volunteers from the local community were recruited via advertising posters. Three participants were excluded based on the exclusion criteria before the start of the study. From the total of 45 volunteers, 15 participants (8 men and 7 women, aged 22.00 ± 2.07 years) with flexible flatfoot who met the inclusion criteria were assigned to the flexible flatfoot group. The inclusion criteria were following:

(1) age 20 to 30 years and
(2) ND more than 10 mm.

The exclusion criteria were:

(1) pain in the lower extremity joints,
(2) other deformities such as tarsal coalition and vertical talus,
(3) any history of surgery involving both lower extremities,
(4) overweight and obesity, and
(5) neuromuscular and neurological disorders.

From the remaining 30 participants, 15 healthy participants with normal feet were age matched and (7 men and 8 women, aged 22.13 ± 1.55 years) were randomly assigned to the normal foot group. The inclusion criteria were as follows:

(1) 20 to 30 years old
(2) ND 5–9 mm.

**Figure 1:**
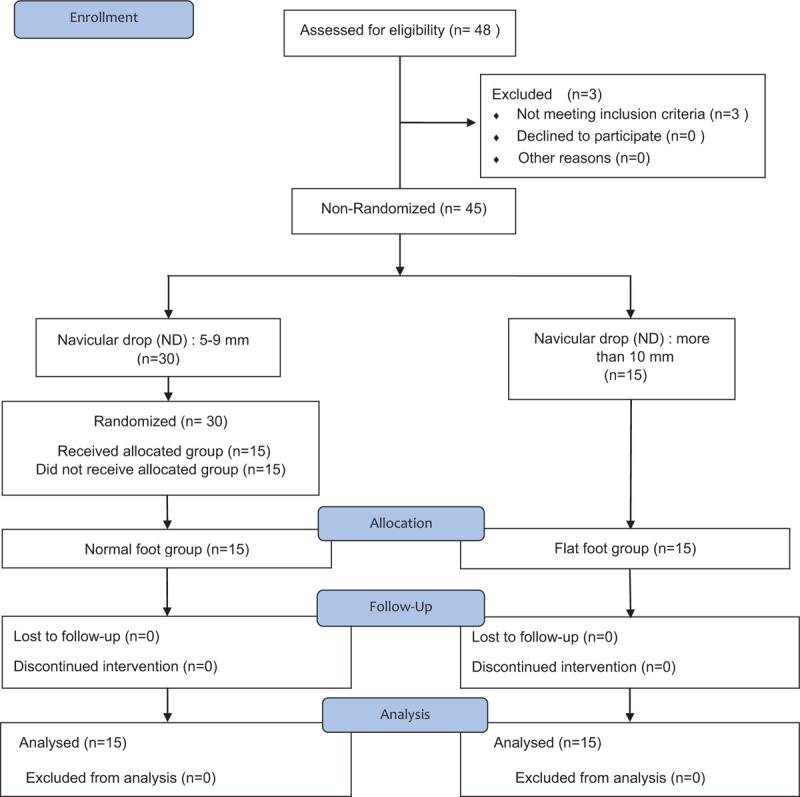
Flow diagram of study participants. The subjects were divided into normal foot group (n = 15) and flexible flat foot group (n = 15) through Navicular drop test. There were no dropouts during the study, and finally 30 subjects participated in the experiment.

Exclusion criteria were the same as that for the flexible flatfoot group (Fig. 1).

Sample size was calculated using G∗power 3.1.9.4 (Franz Faul, Kiel, Germany) based on our previous experimental data. A required sample size of 12 was determined by calculating an estimated effect size of 1.89, alpha level of 0.05, and power of 0.80. Consequently, a total of 30 individuals (15 in each group) were recruited.^[17]^

All subjects understood the experimental procedures and provided written, informed consent prior to participation.

### 2.3 Blinding

It was not possible to blind participants and measurers, given the nature of the exercise and evaluation.

### 2.4 Measurement

#### 2.4.1 ND test

The ND test was performed to confirm whether subjects had flexibility flatfoot. The examiner measured the height of the navicular tuberosity from the ground with the subject being non-weight bearing. Subjects then stood, with weight bearing equally on both feet, as the examiner remeasured the height of the navicular tuberosity. The difference in the height of the navicular tuberosity between weight bearing and non-weight bearing situations was determined. A difference of 10 > mm was considered as flexible flatfoot. ND test have proven valid and reliable for the assessment of the medial arch.^[18]^

#### 2.4.2 Static balance

To determine the static balance, the MatscanVersaTek System (Tekscan Inc., MA) was used to measure the sway area while standing on one leg. The system consists of a HR mat, cuff, USB, and 2-port hub. The HR mat was 0.18-mm thickness and contained 2288 sensors. Data were collected for 30 seconds at 30 frames/second using the Tekscan program, and the sway area was measured using the Research Foot ver. 7.0 (Tekscan Inc., MA). The Matscan System has been validated and shown to be reliable for the static balance test.^[19,20]^

#### 2.4.3 Accuracy of knee joint motions

The accuracy index (AI) of knee joint motions was measured quantitatively by performing tracking tasks of the movement of the knee joint. Two bars were fixed to the both lateral thigh and shin of the subject with a cuff; knee joint flexion and extension were measured by a sensor attached to the lateral side of the knee joint. The degree of motion was measured by attaching a BOURNS 66398S sensor to the knee joint, and data were transmitted to the computer using NI USB-6008 (the National Instruments, TX); error values were calculated using the LabVIEW Ver. 7.0 program (the National Instruments, TX).

AI of the knee joint motions was normalized for each subject's own range of motion, and the differences in tracking target deviation in subjects were measured.^[21]^ AI was quantified using the following formula: 
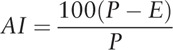


The *P* value was measured as the root mean square (RMS) value between the vertical lines at the upper and lower apexes of the sine wave; the *E* value was calculated as the RMS error between the sine wave line and subject's movement. The size of *P* was based on the scale of the vertical axis, defined as the range of motion of the knee in each subject.

With respect to the tracking task, subjects were instructed to follow the red target baseline proceeding to the sine wave displayed on the computer screen as accurately as possible using the knee flexion and extension motions. The angle of the knee joint that was moved while tracking was between 90° and 180°. Each tracking task was performed for 15 seconds, and the accuracy of the knee joint motions was measured as the average of 3 repeated measurements. The tracking tasks were performed in both the open kinetic chain and closed kinetic chain. The open kinetic chain task was performed sitting in a highchair to make the subjects non-weight bearing. The closed kinetic chain task was performed by flexing and extending the knee repeatedly while bearing the weight with fixed feet.

### 2.5 SFE

**Figure 2:**
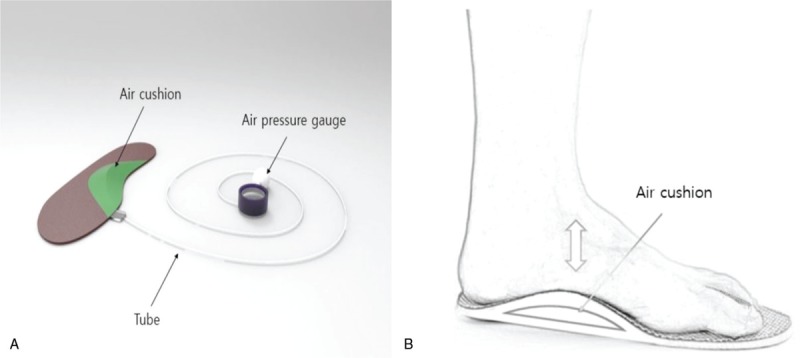
(A) Exercise insole components. (B) Application of short foot exercise.

To provide visual feedback during SFE, a custom-made insole consisting of the insole, tube, and pressure gauge, including an air cushion, was used. Air was inserted into the air cushion located in the MLA when performing SFE to maintain constant exercise while observing the pressure gauge (Fig. 2).

The SFE was divided into 5 steps, and 5-days a week and 20-minute sessions were performed for 5 weeks. At each step, the starting pressure was set to 20 mmHg in the air cushion in the sitting position without a backrest. Subjects were then asked to stand up for the starting posture, and the pressure in the air cushion raised according to the subject's weight. Subjects were asked to reduce and hold the pressure by lifting the MLA as much as possible. SFE were performed at each step in the following positions: standing position, standing on one leg, standing on one leg on an unstable surface, stepping forwards and backwards, and in the squatting position (Fig. 2).

At each step, SFE was performed for 5 seconds holding MLA and another 5 seconds of resting. Three sets were performed, with 1-minute rest in between each set. The left and right feet were exercised alternatingly, and the exercises were performed twice for each foot for a total of 20 minutes. The sequences were randomly selected.

### 2.6 Statistical analysis

Statistical analysis was performed using SPSS 23.0 for Windows. The normality test used the Kolmogorov-Smirnov test. The general characteristics of the subjects were descriptive statistics. A paired *t* test was used to compare between pre- and post-exercise in each group, and an independent *t* test was conducted to test the general characteristics and exercise effects on change scores by subtracting the baseline value from post value between the 2 groups. Statistical significance was α = 0.05.

## 3 Results

### 3.1 Subjects general characteristics

The general characteristics of all subjects are summarized in Table 1. There were no significant difference in the general characteristics, except for the ND test results, between the flatfoot and normal foot groups (Table 1).

**Table 1:**
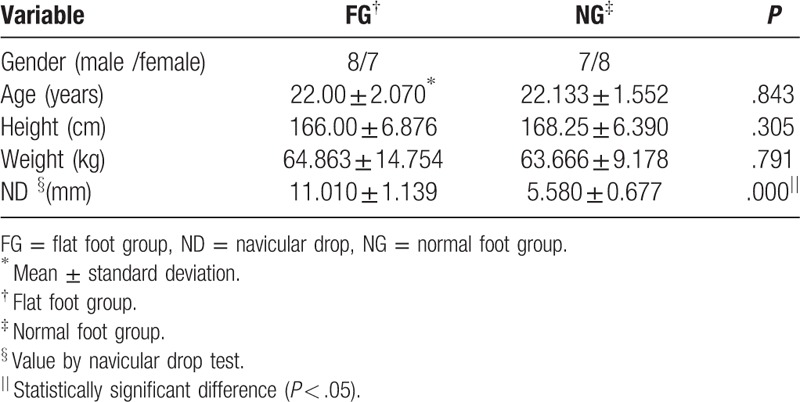
General characteristics and homogeneity of the subjects.

### 3.2 Static balance

**Figure 3:**
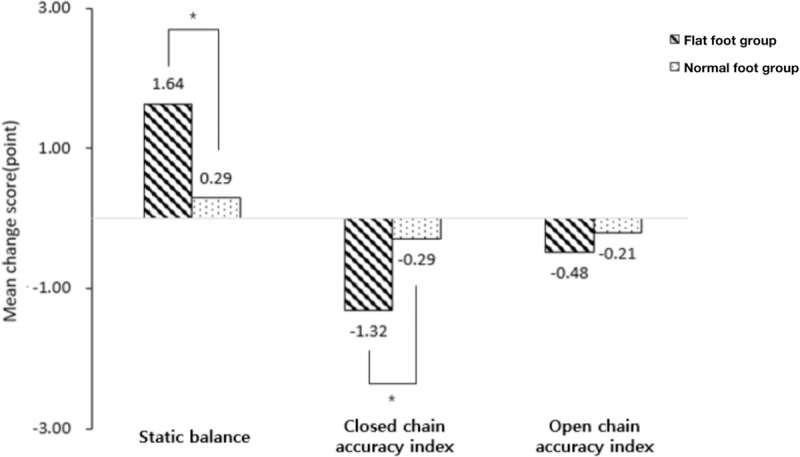
Comparison of pre-post changes between the groups. In static balance, there was a significant difference between the groups. There was a significant difference between the groups in the closed chain. However, there was no significant difference in the open chain. ^∗^*P* < .05.

The static balance was significantly different pre- and post-exercise in the flatfoot group (*P* < .05) (Table 2). However, there was no significant difference pre- and post-exercise in the normal foot group (*P* > .05) (Table 2). There was a significant difference in the change in scores pre- and post-exercise between the 2 groups (*P* < .05) (Table 2; Fig. 3).

**Table 2:**
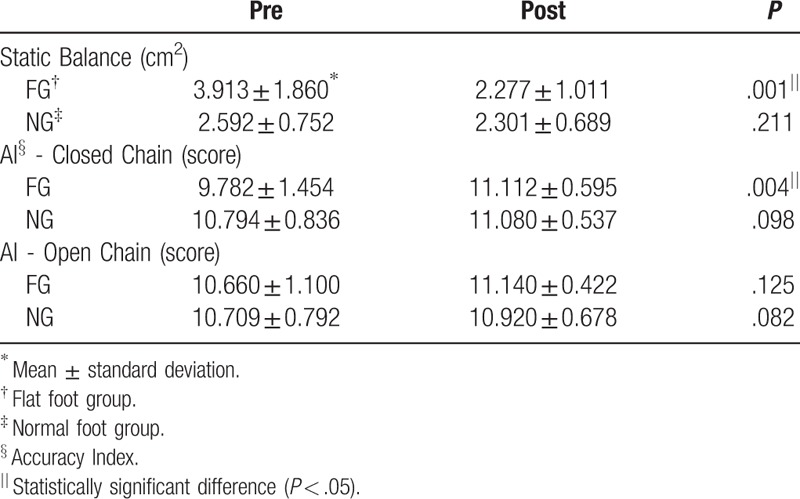
Comparison of the difference between pre and post exercise for each measurement.

### 3.3 Accuracy of knee joint motions

In the flatfoot group, the accuracy of knee joint motions was significantly different between the pre- and post-exercise in the closed chain (*P* < .05) (Table 2) but not in the open chain (*P* > .05) (Table 2). However, in the normal foot group, there was no significant difference between pre- and post-exercise with respect to the accuracy of knee joint motions in the closed and open chain (*P* > .05) (Table 2). Additionally, there was a significant difference in the change scores pre- and post-exercise between the two groups in the closed chain (*P* < .05) but not in the open chain (*P* > .05) (Table 2; Fig. 3).

## 4 Discussion

Flatfoot may cause abnormal descend of MLA, pronation of the heel, and hypermobility of the middle part of the foot, leading to functional instability, pain, posture change, and imbalance.^[3,4,22]^ In this study, we examined the influence of SFE using visual feedback on the balance and accuracy of knee joint movement. Our results show that, while SFE enhanced the balance and accuracy of closed chain knee joint movement in the flatfoot group, SFE showed no training effect in the normal foot group. In the open chain knee joint movement, SFE had no training effects in both, the flatfoot group and the normal foot group. These results are suggestive that SFE using visual feedback could help to improve the knee joint functions in subjects with flat feet.

Intrinsic muscle exercises, such as SFE, play an important role in static balance, such as standing on one leg, and the foot strength is a crucial in adjusting posture.^[23]^ Goldmann et al investigated the effects of toe flexion exercises in normal subjects, and showed that they lead to improvements in walking, running, and jumping.^[24]^ Research conducted by Kelly et al, showed that the activation of AbdH was greater in whilst standing on 1 leg rather than standing on both legs, and that higher level of coordination occurred with other surrounding muscles during medial-lateral sway.^[25]^ Previous studies support our findings with respect to the influence of SFE using visual feedback on static balance.

According to Graham et al, the stabilization of the distal segment in closed chain exercise reduces the moving proximal joint's sear force in providing stability in proximal joint's movement.^[23]^ Especially, squatting is among closed chain exercises, which involves flexion and extension of the ankle joint, knee joint, and hip joint, requiring inter-contraction of neighboring muscles and stability in the distal segments, such as foot and ankle.^[26,27]^ However, weight bearing in flat feet patients increases pronation of the subtalar joint, induces drop of navicular, and lowers stability of foot with descending MLA.^[28]^ In the closed chain exercise for the knee joint, the accuracy of knee joint motion was improved as the SFE contributed to foot stability and mutual coordination with the neighboring muscles. In contrast, the SFE showed no significant increase in the accuracy for open chain exercise because open chain exercise is not related to the stability of ankles.

Static balance and accuracy of knee movement in the closed chain showed a significant difference between the 2 groups. Flatfoot causes MLA to descend, and pronation of calcaneus leads to weaker intrinsic muscles, posture control, and balancing.^[4,23,25]^ SFE effectively strengthens the intrinsic muscles, such as AbdH, which may play an important role in supporting MLA. Jung et al, compared the muscle activation of AbdH between toe curling and SFE.^[29]^ They found that SFE induced greater muscle activation, suggesting that it may prevent deformations, such as flatfoot. The aforementioned previous studies showed that SFE effectively strengthens the weak intrinsic muscles in the flatfoot group to enhance weight support and stability. Thus, the use of visual feedback caused a significant change in the flatfoot group, illustrating a positive influence on the improvement of functions of the flatfoot group in this study. Furthermore, our results show that SFE had no effect in the normal group due to ceiling effect.

This study also provided visual feedback for reducing compensation movement and facilitating accurate movement during SFE. Okamuraa et al compared AbdH activation in general SFE and SFE using visual feedback using a diode light gauge.^[30]^ They found that SFE using visual feedback induced more muscle activation. In our finding, it is reasonable to suggest that SFE using visual feedback may influence on the static balance and distal joint stability.

In conclusion, this study examined the influence of SFE using visual feedback on the balance and accuracy of knee joint movement in subjects with flatfoot and found that SFE using visual feedback improved the balance and accuracy of knee movement. To the best of our knowledge, this is the first study that identified the effectiveness of the SFE program by using visual feedback using pressure gauges during exercise performance. Clinically, visual feedback may be provided during SFE to facilitate the accuracy of the exercise. The limits of the current study are as follows. First, a small sample size limits the interpretation and generalizability of our findings. Secondly, accurate imaging analysis was unavailable in measuring the descending of the navicular bone. Thirdly, this study measured the quantitative intensity of SFE. Fourthly, this study did not compare the effects of SFE only without visual feedback. Thus, further studies are required to investigate the effects of visual feedback on the SFE program.

## Author contributions

**Conceptualization:** Mi Young Lee.

**Data curation:** Ju Sang Kim.

**Formal analysis:** Ju Sang Kim, Mi Young Lee.

**Methodology:** Ju Sang Kim, Mi Young Lee.

**Resources:** Mi Young Lee.

**Supervision:** Mi Young Lee.

**Writing – original draft:** Ju Sang Kim, Mi Young Lee.

**Writing – review & editing:** Ju Sang Kim, Mi Young Lee.
